# Dapagliflozin versus saxagliptin as add-on therapy in patients with type 2 diabetes inadequately controlled with metformin

**DOI:** 10.20945/2359-3997000000056

**Published:** 2018-08-01

**Authors:** Julio Rosenstock, Chantal Mathieu, Hungta Chen, Ricardo Garcia-Sanchez, Gabriela Luporini Saraiva

**Affiliations:** 1 Dallas Diabetes Research Center Dallas TX USA Dallas Diabetes Research Center at Medical City, Dallas, TX, USA; 2 UZ Leuven Leuven Belgium UZ Leuven, Leuven, Belgium; 3 AstraZeneca Gaithersburg MD USA AstraZeneca, Gaithersburg, MD, USA

**Keywords:** Dapagliflozin, type 2 diabetes, SGLT2 inhibitor

## Abstract

**Objective:**

This analysis compared the efficacy and safety of the sodium-glucose cotransporter-2 (SGLT2) inhibitor, dapagliflozin, and the dipeptidyl peptidase-4 (DPP4) inhibitor, saxagliptin, both added on to metformin.

**Materials and methods:**

This was a post-hoc analysis from a double-blind, randomized, 24-week clinical trial (NCT01606007) of patients with type 2 diabetes (T2D) inadequately controlled with metformin. We compared the dapagliflozin 10 mg (n = 179) and saxagliptin 5 mg (n = 176) treatment arms.

**Results:**

Dapagliflozin showed significantly greater mean reductions versus saxagliptin in HbA_1c_ (difference versus saxagliptin [95% CI]: −0.32% [-0.54, −0.10]; p < 0.005), fasting plasma glucose (-0.98 [-1.42, −0.54] mmol/L; p < 0.0001), body weight (-2.39 [-3.08, −1.71] kg; p < 0.0001) and systolic blood pressure (SBP) (-3.89 [-6.15, −1.63] mmHg; p < 0.001). More dapagliflozintreated than saxagliptin-treated patients achieved the composite endpoint of HbA_1c_ reduction ≥ 0.5%, weight loss ≥ 2 kg, SBP reduction ≥ 2 mmHg and no major/minor hypoglycemia (24% versus 7%). No major events of hypoglycemia were reported. More patients on dapagliflozin (6%) versus saxagliptin (0.6%) experienced genital infections.

**Conclusion:**

Dapagliflozin demonstrated greater glycemic efficacy than saxagliptin with additional benefits on weight and SBP, and the safety profile was consistent with previous studies.

## INTRODUCTION

Type 2 diabetes (T2D) is a progressive disease, requiring treatment intensification over time in order to maintain glycemic control. Metformin is the gold standard first-line pharmacological treatment (1), and thereafter as glycemia worsens, sequential add-on treatment decisions are based on a number of variables and usually involve the addition of other oral glucoselowering agents (2). Recommendations from clinical guidelines on the management of T2D vary, with some making no specific recommendations on the choice of add-on agent (1), and others advocating a hierarchical order for add-on therapies (3).

The position statement of the American Diabetes Association (ADA) and European Association for the Study of Diabetes (EASD) recommends the use of one of six commonly used antihyperglycemic agents i.e. 1) a sulfonylurea, 2) a thiazolidinedione, 3) a dipeptidyl peptidase-4 (DPP4) inhibitor, 4) a sodium-glucose cotransporter-2 (SGLT2) inhibitor, 5) a glucagon-like peptide-1 (GLP-1) receptor agonist, or 6) a basal insulin analogue, as an add-on therapy when the individualized HbA_1c_ target is not achieved after ~3 months of treatment with metformin alone (1). The ADA/EASD position statement does not recommend any specific preference for a drug to be used as a dual therapy and instead suggests that the drug choice should be individualized based on patient preferences, hypoglycemia risk, side effect profile, and cost, in addition to other patient and disease characteristics (2). The ADA/EASD position statement further details that other drugs such as α-glucosidase inhibitors, colesevelam, bromocriptine and pramlintide may be used in specific situations but are generally not preferred due to their modest efficacy, side-effect profiles and dosing frequency.

DPP4 inhibitors and SGLT2 inhibitors are widelyused therapies for T2D that are associated with a low incidence of hypoglycemia, with DPP4 inhibitors being body-weight neutral (4) and SGLT2 inhibitors promoting weight loss and also reducing systolic blood pressure (SBP) (5-8). It is worth noting that in the 2017 American Association of Clinical Endocrinologists (AACE)/American College of Endocrinology (ACE) comprehensive glycemic control algorithm, SGLT2 inhibitors have been placed before DPP4 inhibitors in the hierarchical order of recommended use as monotherapy as well as add-on therapy (3).

The SGLT2 inhibitors, canagliflozin and empagliflozin, have both been directly compared with a DPP4 inhibitor in prospective randomized controlled trials (9-12). Long-term treatment with empagliflozin 25 mg showed greater reductions in HbA1c, body weight and SBP in comparison with sitagliptin 100 mg (9,10). Canagliflozin 300 mg as add-on to metformin, or metformin and a sulfonylurea, reduced both HbA_1c_ and weight to a greater extent than sitagliptin 100 mg (11,12).

However, to date, no large randomized controlled trial has directly compared the SGLT2 inhibitor dapagliflozin with a DPP4 inhibitor. One previously published study compared the combination of dapagliflozin plus saxagliptin with either drug individually, as add-on therapy in patients inadequately controlled on metformin (≥ 1500 mg/d) (13). This study was powered at 90% to detect a 0.4% difference in HbA_1c_ between the group receiving dapagliflozin plus saxagliptin and the groups receiving dapagliflozin or saxaglitpin individually, but was not powered for comparison of the groups receiving dapagliflozin or saxaglitpin individually. To provide clinicians with research evidence on the use of dapagliflozin compared with a DPP4 inhibitor to incorporate into their professional judgment and clinical decision making, a *post-hoc* analysis of the aforementioned study was conducted to compare the efficacy and safety of the two agents, dapagliflozin and saxagliptin, individually added on to metformin (14). It should be noted that saxagliptin and sitagliptin have demonstrated similar efficacy and safety profiles in a head-to-head noninferiority trial (15) and a systematic review (16).

## MATERIALS AND METHODS

### Study design

This was a *post-hoc* analysis of 24-week data from a randomized, three-arm, double-blind, active-controlled Phase 3 study (NCT01606007) (13). Patients with T2D were included in the original study if they were aged ≥ 18 years, had inadequate glycemic control (HbA_1c_ ≥ 8.0% and ≤ 12.0%), were on stable metformin therapy (≥ 1500 mg/day) for ≥ 8 weeks before screening and had a BMI ≤ 45.0 kg/m^2^. The exclusion criteria were pregnancy, uncontrolled hypertension (SBP ≥ 160 mmHg and diastolic blood pressure ≥ 10 mmHg), fasting plasma glucose (FPG) ≥ 270 mg/dL during the 4-week lead-in period, cardiovascular disease (CVD) within 3 months of screening, congestive heart failure (New York Heart Association functional class IV), an estimated glomerular filtration rate < 60 mL/min/1.73 m^2^ or serum creatinine ≥ 1.5 mg/dL in men or ≥ 1.4 mg/dL in women, significant hepatic disease, or having received an antidiabetic medication other, having metformin for more than 14 days during the 12 weeks before screening. Patients initially underwent a 4-week lead-in period during which they received metformin extended release (1500-2000 mg/day) before being randomized 1:1:1 to receive dapagliflozin plus saxagliptin, dapagliflozin plus placebo or saxagliptin plus placebo, in addition to metformin.

Primary and secondary efficacy endpoints were re-analyzed to evaluate the efficacy and safety of dapagliflozin versus saxagliptin as add-on to metformin.

The study was designed and performed in accordance with the ethical principles of Good Clinical Practice as defined by the International Conference on Harmonisation and the Declaration of Helsinki. Institutional review boards or independent ethics committees approved each protocol, and each patient provided written informed consent.

### Outcomes measured

The primary endpoint was change from baseline to 24 weeks in HbA_1c_. Secondary endpoints were changes from baseline to 24 weeks in body weight, FPG levels, and 2-hour postprandial glucose (2-hr PPG) levels (from a liquid meal tolerance test). Composite efficacy endpoints were assessed as (1) HbA1c reduction ≥ 0.5% + body weight reduction ≥ 2 kg; (2) HbA1c reduction ≥ 0.5% + SBP reduction ≥ 2 mmHg; (3) HbA_1c_ reduction ≥ 0.5% + body weight reduction ≥ 2 kg + no major/minor hypoglycemia; and (4) HbA1c reduction ≥ 0.5% + body weight reduction ≥ 2 kg + SBP reduction ≥ 2 mmHg + no major/minor hypoglycemia.

Safety assessments included overall adverse events (AEs), hypoglycemia and AEs of special interest including urinary tract and genital infections. Hypoglycemic episodes were classified as minor (symptomatic or asymptomatic with plasma glucose concentration < 63 mg/dL, regardless of need for external assistance) and major (symptomatic requiring third-party assistance due to severe impairment in consciousness or behavior, with or without plasma glucose concentration, < 54 mg/dL, and prompt recovery after glucose or glucagon administration). Change from baseline to 24 weeks in SBP was recorded as a safety assessment.

### Statistical analysis

Statistical methods used in this post-hoc analysis were the same as those used in the primary analysis of the study (13). Adjusted mean change from baseline value and 95% CI were derived using the longitudinal repeated measures mixed model with nominal p-values. Composite endpoints were derived using logistic regression with the adjustment for baseline parameter (as applicable, depending on the outcome included in each individual composite endpoint). AE data were summarized using descriptive statistics.

## RESULTS

### Patient population

Patient demographics and baseline characteristics were balanced across treatment groups. Full baseline characteristics for the dapagliflozin and saxagliptin dose groups have been published previously (13). In brief, mean age was 54 years and mean duration of T2D was 7.6 years. Mean baseline HbA1c was 8.9% (73 mmol/mol) in the dapagliflozin group and 9.0% (75 mmol/mol) in the saxagliptin group. It should be noted that patients enrolled in this study had a high mean baseline HbA_1c_ (8.9%), with 45% having a baseline HbA_1c_ ≥9.0% (13). Mean baseline BMI was 31.5 kg/m^2^ in the dapagliflozin group and 31.8 kg/m^2^ in the saxagliptin group.

### Efficacy outcomes

**Figure 1:**
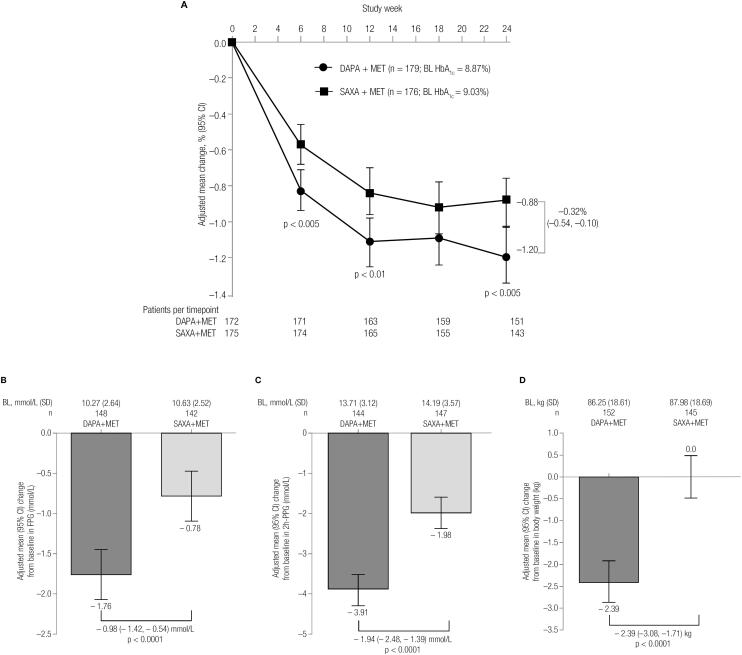
(**A**) Mean change in HbA_1C_ over time in the 24-week treatment period and mean change in (**B**) FPG, (**C**) 2-h PPG, and (**D**) body weight at 24 weeks n is the number of randomized patients with non-missing baseline and Week 24 LOCF values; p-values are for the difference between treatment groups. BL: baseline; DAPA: dapagliflozin; Diff: difference; MET: metformin; SAXA: saxagliptin; CI: confidence interval; FPG: fasting plasma glucose; 2-hr PPG: 2-hour postprandial glucose.

As an add-on to metformin, both dapagliflozin and saxagliptin reduced HbA1C over the 24-week treatment period (Figure 1A). Dapagliflozin showed a greater adjusted mean reduction from baseline in HbA_1c_ over the 24-week period (-1.20% [95% CI: −1.35, −1.04]) versus saxagliptin (-0.88% [-1.03, −0.72]), with a significant mean difference of −0.32% [-0.54, −0.10]; p < 0.005). The significant differences in adjusted mean change in HbA_1c_ with dapagliflozin versus saxagliptin were observed as early as Week 6 (-0.83% [-0.94, −0.71] versus −0.57% [-0.68, −0.46], respectively; p < 0.005). At 24 weeks, dapagliflozin reduced FPG (mean difference [95% CI]: −0.98 mmol/L [-1.42, −0.54]; p < 0.0001; Figure 1B), 2-hr PPG (mean difference [95% CI]: −1.94 mmol/L [-2.48, −1.39]; p < 0.0001; Figure 1C), and body weight (mean difference [95% CI]: −2.4 kg [-3.1, −1.7]; p < 0.0001; Figure 1D) to a significantly greater extent than saxagliptin.

After 24 weeks, the adjusted mean (95% CI) % proportion of patients achieving an HbA_1c_ < 7% was 22.2% (16.1%, 28.3%) with dapagliflozin versus 18.3% (13.0%, 23.5%) with saxagliptin (difference: −4.0% [-11.8%, 3.9%]; p = 0.32). A greater proportion of dapagliflozin-treated versus saxagliptin-treated patients achieved the composite endpoint of HbA_1c_ reduction ≥ 0.5%, weight loss ≥ 2 kg, SBP reduction ≥ 2 mmHg and no major/minor hypoglycemic events (Table 1). Likewise, greater proportions of dapagliflozintreated versus saxagliptin-treated patients achieved other composite endpoints (Table 1). Supplementary Table 1 shows the proportion of patients falling into the different categories according to HbA_1c_ decrease of < or ≥ 0.5% and weight loss of < or ≥ 2 kg.

**Table 1:** Composite efficacy endpoints at 24 weeks

Composite efficacy endpoints	DAPA+MET (n = 179)	SAXA+MET (n = 176)
HbA_1c_ reduction ≥ 0.5% + body wt. reduction ≥ 2 kg + SBP reduction ≥ 2 mmHg + no major/minor hypoglycemia		
Adjusted* % (95% CI)	24.3% (18.0, 30.7)	7.0% (3.3, 10.7)
Adjusted % diff. for SAXA+MET vs DAPA+MET (95% CI for diff.)	17.3% (10.0, 24.7)	
HbA_1c_ reduction ≥ 0.5% + body wt. reduction ≥ 2 kg		
Adjusted* % (95% CI)	38.2% (31.1, 45.4)	11.7% (6.9, 16.4)
Adjusted % diff. for SAXA+MET vs DAPA+MET (95% CI for diff.)	26.6% (18.0, 35.1)	
HbA_1c_ reduction ≥ 0.5% + SBP reduction ≥ 2 mmHg		
Adjusted* % (95% CI)	40.9% (33.7, 48.0)	27.8% (21.4, 34.1)
Adjusted % diff. for SAXA+MET vs DAPA+MET (95% CI for diff.)	13.1% (3.7, 22.5)	
HbA_1c_ reduction ≥ 0.5% + body wt. reduction ≥ 2 kg + no major/minor hypoglycemia		
Adjusted* % (95% CI)	37.7% (30.6, 44.8)	11.7% (6.9, 16.4)
Adjusted % diff. for SAXA+MET vs DAPA+MET (95% CI for diff.)	26.0% (17.5, 34.5)	

n is the number of randomized subjects who received at least one dose of double-blind medication during the treatment period.

* Adjusted for baseline HbA_1c_.

DAPA: dapagliflozin; diff.: difference; MET: metformin; SAXA: saxagliptin; SBP: systolic blood pressure; wt.: weight.

### Safety

The incidence of AEs was similar between groups, and was described in detail in the main publication (13). The proportion of patients with ≥ 1 AE was 49% for dapagliflozin and 53% for saxagliptin (Supplementary Table 2). Hypoglycemic event rates were low and similar across treatment groups (2 [1%] patients in each group). No major hypoglycemia events were reported. Higher proportions of patients on dapagliflozin (10 [6%]) versus saxagliptin (1 [0.6%]) experienced genital infections. No cases of diabetic ketoacidosis, postural hypotension, amputation or hospitalization for heart failure were reported in this study.

A statistically significant reduction in SBP from baseline to 24 weeks was observed with dapagliflozin (mean [95% CI]: −3.3 [-5.0, −1.7] mmHg) versus a small numerical increase observed with saxagliptin (mean [95% CI]: +0.5 [-1.1, 2.1] mmHg; p = 0.0008).

Small increases in total cholesterol of 3.8% (1.5%, 6.3%) and HDL-cholesterol of 7.7% (5.2%, 10.2%) and a non-significant increase in LDL-cholesterol of 1.5% (-3.1%, 6.4%]) were seen in patients receiving dapagliflozin. No significant changes in fasting lipids were seen in patients receiving saxagliptin.

## DISCUSSION

Early combination therapy using agents with complementary mechanisms of action is recommended for advancing glucose control (1). Currently, two pharmacologic classes, DPP4 inhibitors and SGLT2 inhibitors, show promise as preferred second-line treatment options given their weight neutral and weight lowering effects, respectively, and low potential for hypoglycemia. Hence, the comparison between dapagliflozin and saxagliptin as add on to metformin is important in this context.

Many patients with T2D are affected by multiple co-morbidities, including obesity, dyslipidemia, and hypertension, all of which are well documented risk factors for CVD. In our study, dapagliflozin reduced glycemic parameters including HbA1c, FPG and 2-hr PPG to a significantly greater extent than saxagliptin. Consistent with its glycosuric mechanism, dapagliflozin also reduced body weight and SBP versus saxagliptin. Weight reduction with dapagliflozin may benefit overweight/obese patients with T2D. Reduction in SBP with dapagliflozin is also an added benefit in view of the high prevalence of hypertension in patients with T2D. These results are consistent with other studies of SGLT2 inhibitors versus DPP4 inhibitors (10,11). Such findings may have contributed to the placement of SGLT2 inhibitors in the AACE/ACE comprehensive glycemic control algorithm, suggesting that SGLT2 inhibitors are an important addition for individualizing the treatment of T2D.

The EMPA-REG OUTCOME trial showed that patients with T2D and established CVD disease who received empagliflozin versus placebo, had a lower rate of the primary composite of death from cardiovascular (CV) causes, non-fatal myocardial infarction or nonfatal stroke with substantial reductions in CV mortality when the study drug was added to standard care (17). The results from the CANVAS study further support the effects of SGLT2 inhibitors on CV outcomes, with a significantly lower rate of the composite outcome of death from CV causes, non-fatal myocardial infarction or non-fatal stroke in the canagliflozin group, and also for the exploratory outcomes of hospitalization for heart failure, although canagliflozin did not show a significant reduction on CV mortality (18). More recently, real-world observational evidence has suggested a beneficial class effect of SGLT2 inhibitors (canagliflozin, dapagliflozin and empagliflozin) on CV outcomes (19,20), and findings from the ongoing CV study DECLARE (NCT01730534) may further establish this possible class effect. The CV outcomes trials of DPP4 inhibitors have shown no beneficial effects of DPP4 inhibitors on CV outcomes; however, no increase in the risk of major adverse CV events, such as the combined MACE outcome (CV death, nonfatal myocardial infarction and non-fatal stroke), versus standard care was noted.

As expected, both dapagliflozin and saxagliptin were well tolerated in the current study. Higher proportions of patients on dapagliflozin versus saxagliptin reported genital infections, consistent with previous observations from the dapagliflozin clinical trial program (21). There was a low incidence of hypoglycemia in both treatment groups, which reflects the self-limiting modes of action of these agents, i.e. reduction in the amount of filtered glucose with decreasing blood glucose levels causes a low potential for hypoglycemia with dapagliflozin; and glucose-stimulated insulin secretion decreases in patients treated with saxagliptin as physiological glucose levels are approached, leading to a low risk of hypoglycemia.

A limitation of this study is that it is a *post-hoc* analysis, and should be regarded as hypothesis-generating. These data are still of scientific value as findings from this study are similar to the previously reported data from comparisons between other SGLT2 inhibitors and DPP4 inhibitors in randomized controlled trials (11,12).

In conclusion, in this post-hoc analysis, addition of dapagliflozin or saxagliptin to metformin improved glycemic control in patients poorly controlled with metformin. However, significantly greater glycemic efficacy was observed with dapagliflozin compared with saxagliptin, together with reductions in body weight and blood pressure which may provide additional benefit to many patients with T2D. The safety profiles of both treatments were consistent with previous observations in their respective clinical trials.

## Appendix

**Supplementary Table 1:** Unadjusted changes in HbA_1c_ and weight at 24 weeks

DAPA + MET	HbA_1c_ decrease of ≥ 0.5%	HbA_1c_ decrease of < 0.5% or HbA1c increase
Weight decrease of ≥ 2 kg	37.6%	9.8%
Weight decrease of < 2 kg or weight increase	34.7%	17.9%
SAXA + MET	HbA_1c_ decrease of ≥ 0.5%	HbA_1c_ decrease of < 0.5% or HbA_1c_ increase
Weight decrease of ≥ 2 kg	12.0%	7.4%
Weight decrease of < 2 kg or weight increase	54.9%	25.7%

Data are proportions of patients (%). DAPA: dapagliflozin; MET: metformin; SAXA: saxagliptin.

**Supplementary Table 2:** Proportions of patients experiencing adverse events over 24 weeks

n (%)		DAPA + MET (n = 179)	SAXA + MET (n = 176)
≥ 1 Adverse event		87 (49)	93 (53)
≥ 1 Serious adverse event		2 (1)	6 (3)
Adverse events leading to discontinuation		1 (1)	0
Adverse events of special interest			
	Hypoglycemia*	2 (1)	2 (1)†
	Urinary tract infection	7 (5)	9 (5)
	Genital infection	10 (6)	1 (1)
	Glomerular filtration rate decrease	0	1 (1)

* Hypoglycemia includes minor and major;

† Patients with more than one type of hypoglycemic episode were counted within each category but only once for patients experiencing hypoglycemia; DAPA: dapagliflozin; MET: metformin; SAXA: saxagliptin.
